# SGLT2 inhibitors for alleviating heart failure through non-hypoglycemic mechanisms

**DOI:** 10.3389/fcvm.2024.1494882

**Published:** 2024-12-09

**Authors:** Ya-ru Chen, Fang-yuan Zhu, Rong Zhou

**Affiliations:** Department of Cardiology, The Second Hospital of Shanxi Medical University, Taiyuan, Shanxi, China

**Keywords:** sodium-glucose cotransporter-2 inhibitor, non-diabetic, heart failure, cardiovascular outcome trials, empagliflozin, dapagliflozin

## Abstract

Sodium-glucose cotransporter-2 (SGLT2) inhibitors afford significant cardiovascular benefits to patients with diabetes mellitus and heart failure. Three large randomized clinical trials (EMPAREG-Outcomes, DECLARE-TIMI58, and DAPA-HF) have shown that SGLT2 inhibitors prevent cardiovascular events and reduce the risk of death and hospital admission resulting from heart failure. Patients without type 2 diabetes mellitus (T2DM) also experience a similar degree of cardiovascular benefit as those with T2DM do. SGLT2 inhibitors could improve cardiac function through potential non-hypoglycemic mechanisms, including the reduction of the circulatory volume load, regulation of energy metabolism, maintenance of ion homeostasis, alleviation of inflammation and oxidative stress, and direct inhibition of cardiac SGLT1 receptors and antimyocardial fibrosis. This article reviews the mechanism through which SGLT2 inhibitors prevent/alleviate heart failure through non-hypoglycemic pathways, to support their use for the treatment of heart failure in non-T2DM patients.

## 1 Introduction

Heart failure is a clinical syndrome characterized by fatigue, dyspnea, reduced exercise tolerance, and fluid retention owing to reduced ventricular filling and/or ejection fraction (1). Worldwide, 1%–2% of adults suffer from heart failure. Heart failure leads to reduced quality of life, high morbidity and mortality, and a heavy economic burden (2). An analysis of medical insurance data of 50 million urban workers in six provinces and municipalities directly under the central government in China in 2017 showed that the country has a standardized prevalence rate of 1.1% for heart failure, with an incidence of heart failure of 275/100,000 person-years (287/100,000 person-years for men and 261/100,000 person-years for women). In addition, 3 million new cases of heart failure are being reported per year in the country (3). Despite the application of various active pharmacological and non-pharmacological interventions, the recurrence and mortality rates resulting from heart failure are not optimistic. In May 2020, the FDA approved dapagliflozin for the treatment of heart failure with reduced ejection fraction (HFrEF) in adults with or without T2DM. Sodium-glucose cotransporter-2 (SGLT2), a group of active glucose transporter proteins, is mainly expressed in the kidney (4). SGLT2 transports glucose into the cell through the sodium concentration gradient established by the Na^+^/H^+^-ATPase pump, prompting glucose transporters (GLUTs) to participate in glucose reabsorption by utilizing the diffusion gradient of glucose across the plasma membrane (5). An SGLT2 inhibitor is a novel hypoglycemic agent that exhibits unique non-insulin-dependent hypoglycemic effects, affording cardiovascular benefits by competitively binding SGLT2 protein to glucose, reducing glucose and Na^+^ reabsorption in renal proximal tubules, lowering the renal glycemic threshold, increasing urinary glucose excretion to lower the concentration of glucose in the body, correcting hyperglycemic toxicity, regulating energy metabolism of cardiomyocytes, and attenuating oxidative stress (6). Several recent studies have confirmed that the benefits afforded by SGLT2 in terms of symptomatic relief and prognostic improvement in patients with heart failure far exceed those achieved by improving metabolism through glucose lowering, suggesting that improving symptoms and prognosis in heart failure is a class effect of SGLT2 inhibitors. Hence, the therapeutic potential of SGLT2 inhibitors for nondiabetic heart failure should be further explored (7).

## 2 Reduction in the circulating volume load

Volume overload is an important pathophysiological process in the development of heart failure. In heart failure, myocardial contractile function decreases, which, in turn, decreases the cardiac output. As a result, the body cannot meet its metabolic needs, the effective circulating blood volume decreases, sympathetic excitability increases, and the renin–angiotensin system (RAS) is activated. All these changes lead to compensatory fluid retention and redistribution, an increase in central venous pressure and ventricular filling pressure, and tissue fluid retention in the interstitial spaces of the tissues, which lead to cardiac circulatory dysfunction with dyspnea and bilateral lower extremity edema (8). The main function of SGLT2 inhibitors is to reduce glucose and Na^+^ reabsorption in the renal proximal tubules, which results in the urinary excretion of glucose and Na^+^. Consequently, osmotic diuresis occurs, which decreases the plasma volume (9). The SGLT2-inhibitor monotherapy led to a mean reduction of 3–5 mmHg in systolic blood pressure. A pooled analysis of a series of combination therapies reported a similar reduction in systolic blood pressure (10). Solomon et al. (11) studied 228 cases of uncontrolled hypertension, preserved ejection fraction, and left ventricular diastolic dysfunction (LVDD) and reported that the degree of improvement in left ventricular diastolic function is correlated with the degree of reduction in systolic blood pressure. The greatest improvement in LVDD was seen in patients with the lowest systolic blood pressure after treatment with SGLT2 inhibitors. In a model of nondiabetic obese rats with hypertension (DS/obese rats), treatment with ipragliflozin improved left ventricular hypertrophy as well as changes in the left ventricular wall thickness and fibrosis associated with increased urinary output. In addition, systolic blood pressure was lower in the experimental group of rats than it was in the DS/obese control group at 11 weeks of age. However, the levels of blood glucose and insulin were not impacted (12). Kravtsova et al. (13) showed that the administration of dagliflozin for 3 weeks prevented the progression of salt-induced hypertension in a nondiabetic Dahl salt-sensitive rat model of salt-sensitive hypertension, independent of gender.

It has been proposed that the diuretic effect of SGLT2 inhibitors differs from that of the loop diuretics. A mathematical modeling was conducted that demonstrated that dapagliflozin reduced the interstitial fluid volume twice that of blood volume, whereas bumetanide reduced the interstitial fluid volume reduction by only 78% of the blood volume reduction. Hence, it can be suggested that the main function of SGLT2 inhibitors is to reduce the interstitial fluid volume. As SGLT2 inhibitors can thicken the blood as well as reduce the interstitial fluid volume, they enhance the delivery of oxygen to myocardial tissues (14). This dual effect of SGLT2 inhibitors is responsible for their beneficial effects in heart failure (15). A randomized trial of intensive blood pressure control vs. standard blood pressure control (SPRINT) (16) demonstrated that controlling systolic blood pressure to <120 mmHg, instead of to <140 mmHg, significantly reduced the incidence of cardiovascular events, notably heart failure. In the EMPA-REG OUTCOME (17) and DECLARE-TIMI58 (18) trials, systolic blood pressure in patients' background data was 130–140 mmHg, which SGLT2 inhibitors reduced by approximately 5 mmHg. Hence, it can be suggested that the antihypertensive effect of SGLT2 inhibitors is, at least partly, responsible for suppression of cardiovascular events, particularly heart failure.

## 3 Regulation of energy metabolism

### 3.1 Ketone bodies

Murashige et al. (19) analyzed plasma samples obtained from the radial artery, coronary sinus, and femoral vein of 110 patients including heart failure patients and controls through targeted metabolomics (600 nutrient metabolites) and detected a total of 227 nutrient metabolites in the samples. Of these nutrient metabolites, ketone bodies, acetate, and glutamate were taken up in large amounts in both the heart and legs, with the ketone-body uptake being particularly high in patients with heart failure. Ketone bodies are energetically efficient fuels because they produce large amounts of adenosine triphosphate (ATP) molecules with low oxygen demand (20). Therefore, an increased uptake of ketone bodies is believed to protect against heart failure. A significant correlation between the plasma BNP levels and the cardiac uptake of ketone bodies suggests that cardiac utilization of ketone bodies increases with the deterioration of the left ventricular function (21). It has been shown that advanced stages of heart failure are characterized by the myocardial utilization of ketone bodies, despite impaired ketone-body utilization by skeletal muscles (22).

Ketone bodies not only serve as an energy source but are also involved in intracellular signaling. β-Hydroxybutyric acid is an endogenous inhibitor of histone deacetylase (HDAC). HDAC affects the acetylation state of histones and other important cellular proteins and regulates gene expression, including therapeutic targets for pathological cardiac remodeling (23). Lkhagva et al. (24) examined the effects of HDAC in a rat model of isoproterenol-induced heart failure. They demonstrated that oral HDAC inhibitors reduced the amplitude of the increase in inflammatory factors, reduced ventricular volume and improved cardiac function. Hence, it can be hypothesized that ketone bodies can reduce cardiac inflammation by inhibiting HDAC. β-Hydroxybutyric acid is a ligand for G-protein-coupled receptor 41 (GPR41), which is highly expressed in sympathetic ganglia. Its inhibition by β-hydroxybutyric acid suppresses both the sympathetic nervous system activity and heart rate (25). Hence, it can be suggested that ketone bodies directly modulate sympathetic nerve activity, thereby controlling the energy expenditure required for maintaining metabolic homeostasis, which may ameliorate heart failure.

### 3.2 Binding of SGLT2 inhibitors to glucose transport proteins

Li et al. (26) showed that, in advanced stages of heart failure, where increased glycolysis adversely affects mitochondrial uncoupling, oxidative phosphorylation, and cardiac energy supply, the binding of empagliflozin to GLUT1 and GLUT4 may help slow down the progression of the disease. Mustroph et al. (27) investigated the direct metabolic effects of SGLT2 inhibitors on cardiomyocytes and demonstrated that when isolated resting cardiomyocytes obtained from human end-stage heart failure transplanted hearts or transverse aortic constriction (TAC)-induced heart failure mouse hearts were treated with 1 μM of empagliflozin for 24 h in the presence of albumin, the expression of the glucose transporter protein GLUT1 increased. However, this effect was not observed in GLUT4.

### 3.3 SGLT2 inhibitors and lipid metabolism

SGLT2 inhibitors stimulate lipolysis, lipid oxidation, and ketogenesis, all of which reduce body fat (28). Sawada et al. (29) studied the anti-obesity mechanism of SGLT2 inhibitors. They divided diet-induced obese mice into two groups: experimental and control. Then, they subjected the experimental animals to hepatic vagotomy (HVx) and the control animals to a sham operation. Both animal groups were then fed a high-fat diet containing 0.015% tofogliflozin for 3 weeks. In contrast to the sham-operated control group, the HVx group demonstrated reduced adiposity and attenuated levels of protein kinase A (PKA) phosphorylation in their white adipose tissues. This is because the PKA pathway can act as an effector of the liver–brain–adipose axis and activate triglyceride lipase in adipocytes. Yang et al. (30) experimentally demonstrated that canagliflozin promotes fat thermogenesis and catabolism via the β-adrenergic receptor-cyclic adenosine 3′,5′-monophosphate-protein kinase A pathway. These results suggest that SGLT2 inhibition triggers glycogen-depletion signaling and initiates the liver–brain–adipose axis, leading to PKA activation within adipocytes. It can also be suggested that the weight-loss effects of SGLT2 inhibition are mediated, in part, through the liver–brain–adipose neural circuit.

Lee et al. (31) confirmed that the effect of empagliflozin on triglycerides in obese adults varies according to the visceral adipose tissue (VAT). Empagliflozin inhibits the synthesis of triglycerides in subjects with low VAT, whereas it increases triglycerides in subjects with high VAT. Empagliflozin shows a unique effect on lipid metabolism in obese nondiabetic adults, depending on VAT levels in such adults. It inhibits triglyceride synthesis in subjects with low VAT but causes weight loss in those with high VAT even though it increases triglycerides in such individuals. We speculate that the trend toward elevated triglycerides in the latter may be transient and related to VAT mobilization.

## 4 Ion homeostasis

### 4.1 Na^+^/H^+^ exchanger (NHE)

Nakamura et al. (32) demonstrated that NHE1 activation increases intracellular Na^+^ loading, which reduces or reverses the drive for Na^+^/Ca^2+^ exchanger-mediated Ca^2+^ efflux and leads to intracellular Ca^2+^ overload and contractile dysfunction. In experimental models of heart failure, inhibition of NHE has been shown to reduce cardiac hypertrophy, fibrosis, remodeling, and contractile dysfunction (33). Trum et al. (34) reported abundant expression of NHE1 in human atrial and ventricular tissues. They also noted a higher expression of atrial NHE1 in HFpEF and atrial fibrillation compared to that in patients without heart failure, especially in end-stage heart transplant recipients. Significant increases in atrial and ventricular NHE1 expressions were observed in patients with heart failure. The group of Zuurbier were the first showing that SGLT2i's can inhibit the NHE1 and lower intracellular Na+ and Ca2+ in cardiomyocytes from rabbit and mouse (35, 36) and in human endothelial cells (37). Significant inhibition of NHE activity was observed when human atrial myocytes and mouse ventricular myocytes were acutely exposed to empagliflozin (1 μmol/L for 10 min). Baartscheer et al. (35) reported that in the absence of albumin, acute (<10 min) or prolonged (3 h) incubation of ventricular myocytes with empagliflozin (0.2–1.0 µM) reduced the cytoplasmic levels of Na^+^ in healthy rabbit cardiomyocytes. In the absence of albumin, 1 µM of empagliflozin attenuated late I_Na_ in mouse cardiomyocytes with heart failure or sodium-channel mutations (38). Late attenuation of I_Na_ helps to reduce cardiac action potential prolongation and prevent arrhythmias associated with action potential prolongation.

Although most studies could not detect SGLT2 expression in the human heart (39), the aforementioned experimental SGLT2 inhibitors function independently of glucose levels. Hence, it can be suggested that this is a class effect of SGLT2 inhibitors (36). That SGLT2 inhibitors results in cardioprotection independent of SGLT2 was also supported by a study showing that empagliflozin could lower infarct size followinh a cardiac ischemia-reperfusion insulin in wild-type and SGLT2 knockout animals alike (40). Therefore, SGLT2 inhibitors may confer their protective effects by inhibiting the NHE directly in the myocardium, irrespective of SGLT2 inhibition.

### 4.2 Mitochondrial Ca^2+^

Ca^2+^ homeostasis in cardiomyocytes is maintained by the inflow of mitochondrial Ca^2+^ unidirectional (MCU) transport proteins and the outflow of the mitochondrial Na^+^/Ca^2+^ exchanger (mNCE) (41). It has now been demonstrated that an increased expression of NHE1 in the state of heart failure leads to an overload of intracellular Na^+^, which induces a decrease in the [NAD(P)/NAD(P)^+^] redox potential and an increase in oxidative stress. An increase in the concentration of mitochondrial Na^+^ decreases the fluidity of the inner mitochondrial membrane, which promotes the production of mitochondrial ROS (42). In contrast, a decrease in the concentration of mitochondrial Ca^2+^ hinders the tricarboxylic acid-derived production of NADH through the inhibition of dehydrogenase or F0F1 ATP synthase, which leads to energy deficiency and a reduction in the NADPH-generating ability of antioxidants (43). In addition, an elevated concentration of cytoplasmic Na^+^ in the heart leads to a reduction in the concentration of mitochondrial Ca^2+^ and/or an increase in that of cytoplasmic Ca^2+^. Consequently, cardiac metabolism can shift from fatty acid metabolism to glucose metabolism (44). In addition to inhibiting NHE to indirectly increase the concentration of mitochondrial Ca^2+^ (35) to improve cardiac function, SGLT2 inhibitors increase the activity of the sarcoplasmic/endoplasmic reticulum Ca^2+^–ATPase responsible for Ca^2+^ re-uptake (SERCA2a). As a result, LV diastolic function improves (4). Another way in which empagliflozin regulates Ca^2+^ involves the inhibition of calcium/calmodulin-dependent protein kinase II (CaMKII). Using a mouse model, it has been shown that this inhibition slows down calcium loss from the sarcoplasmic reticulum, resulting in stronger myocardial contractions (45).

## 5 Inflammation and oxidative stress

Both oxidative stress and inflammation have significant and interdependent effects on major cardiovascular diseases. Oxidative stress generates large amounts of ROS, which can induce the opening of the mitochondrial transition pore and the overexpression of transcription factors, thereby affecting mitochondrial function (46). Numerous experimental and clinical studies have shown that ROS enhances damage through nonspecific interactions with proteins, lipid membranes, mitochondria, and DNA. Such deleterious effects are relevant in advanced heart failure and in reperfusion after myocardial ischaemia (47). Inflammation is part of the body's immune response to defend itself against pathogens and to clear out infections. However, patients suffering from heart disease usually develop chronic inflammation that impairs their cardiac health, even if they are not showing any signs of infection (48).

### 5.1 Cardiomyocytes

In cardiomyocytes isolated from animals fed a high-fat diet, the use of empagliflozin (1 µM) for 2 h in the absence of albumin reduces the ROS levels in cardiomyocytes under metabolic stress through the activation of AMP-activated protein kinase (AMPK) and sestrin2. Kolijn et al. (49) showed that in patients with HFpEF who have undergone left ventricular biopsies, 1-h treatment with empagliflozin (0.5 µM) significantly attenuated oxidized protein kinase GIα (PKGIα), improved the myofilament protein phosphorylation, decreased the cardiomyocyte stiffness, and reduced the cytokine levels. Liu et al. (50) performed the ligation of the left anterior descending artery in C57BL/6 mice, which induced myocardial infarctions in the mice. The animals were postoperatively randomly assigned to two groups for administering either saline or empagliflozin. Compared to the saline-administered group, the empagliflozin -treated group showed improved cardiac function, reduced infarct size, and a decrease in interstitial fibrosis. Hence, it can be suggested that empagliflozin inhibits cardiomyocyte apoptosis by alleviating oxidative stress and restoring the mitochondrial membrane potential.

### 5.2 Endothelial cells

Various *in vitro* studies using human and murine models have shown that SGLT2 inhibitors exhibit anti-inflammatory effects by directly reducing endothelial cell and macrophage inflammatory molecules. Gaspari et al. (51) were the first to report the direct endothelial effects of SGLT2 inhibitors. They demonstrated that in the presence of albumin, low concentrations of dapagliflozin (<5 nM) decreased the TNFα-induced NF-κB mRNA expression in human umbilical vein endothelial cells. They also reduced the stretch-induced ROS production in human coronary artery endothelial cells. However, this effect was lost with the intervention of the NHE-1 inhibitor cariporide (52). Hence, it can be suggested that the TNFα-induced activation of NHE-1 produces ROS, which increases the intracellular Na^+^ (37). Zhou et al. (53) reported that dapagliflozin can reverse the H_2_O_2_-mediated inhibition of serine phosphorylation of endothelial nitric oxide synthase (eNOS) and the expression of sirtuin 1 (SIRT1) in endothelial cells. The function of such endothelial cells can be improved by restoring the eNOS activity, restoring NO bioavailability, and activating SIRT1. As a result, the ROS production is reduced, which ameliorates the endothelial dysfunction. Heme oxygenase-1 (HO-1) is an endogenous antioxidant enzyme. Peyton et al. (54) treated human endothelial cells with canagliflozin and found that it stimulated a concentration- and time-dependent increase in HO-1, which correlated with a significant increase in HO activity. In addition, they reported that canagliflozin induced a concentration-dependent inhibition of endothelial cell proliferation, DNA synthesis, and migration, independent of the inhibition of HO-1 activity and/or expression.

### 5.3 Smooth muscle cells (SMCs)

Sustained inflammation and oxidative stress result in the onset and progression of various vasoproliferative diseases. Notably, the proliferation and migration of SMCs play a key role in the pathogenesis of these diseases. Sukhanov et al. (55) reported low levels of SGLT2 expression in human aortic SMCs. They also demonstrated that pretreatment of these SMCs with empagliflozin increases the expressions of SGLT2 and NLRP3. In addition, empagliflozin impacts the production of IL-17A/TRAF3IP2-dependent superoxide and hydrogen peroxide, the expression of NLRP3, the activation of caspase-1, and the release of mitogenic and migratory IL-1β and IL-18. All these effects inhibit the proliferation and migration of SMCs at normal glucose levels and without inducing cell death. Benetti et al. (56) found that empagliflozin also inhibits SGLT2 expression by suppressing NLRP1 expression, which, in turn, reduces the levels of pro-inflammatory and pro-atherosclerotic cytokines IL-18β and IL-3 in SMCs.

### 5.4 Cardiac fibroblasts

Yumei et al. (57) reported that dapagliflozin increases P-AMPK in cardiac fibroblasts exposed to lipopolysaccharide. Dapagliflozin attenuates the increase in NHE-1 mRNA and the association between NHE-1 and heat shock protein-70 (Hsp70). This effect depends on the activation of AMPK.

## 6 SGLT1

Sayou et al. (58) conducted their studies on patients with end-stage heart failure undergoing heart transplantation. They found that SGLT1 expression in myocardial left ventricular tissue samples was positively correlated with the left ventricular end-diastolic diameter (LVEDD) and negatively with LV systolic function. However, no SGLT2 expression was detected in the myocardium. A related study explored whether SGLT2 inhibitors have any targets of action in human cardiomyocytes. The SGLT1 and SGLT2 gene expressions were examined in 23 human tissues using real-time quantitative PCR. The results showed that SGLT2 could be barely detected in human and animal hearts, whereas SGLT1 was highly expressed in the myocardium (39). Sayou et al. (59) used TAC, abdominal aortic shunt, and inferior vena cava shunt to induce chronic progressive pressure overload and volume overload in Wistar rats, which led to heart failure in these animals. In addition, they demonstrated that SGLT1 protein expression was significantly up-regulated in the left ventricle of rats. Under glucose or hemodynamic overload, cardiomyocytes show increased expression of SGLT1. This phenomenon promotes the influx of sodium and glucose into the cells, leading to glucose accumulation, ionic disorders, and calcium overload. These effects together accelerate myocardial damage, thereby causing myocardial fibrosis (60). Dasari et al. (61) suggested that glycolipotoxicity mediates oxidative stress, apoptosis, and necrosis through the up-regulation of SGLT1, which then increases cardiomyocyte injuries.

Bode et al. (62) found that long-term treatment with sotagliflozin, a dual SGLT-1 and SGLT-2 inhibitor, was effective in alleviating left atrial cardiomyopathy in a rat model of metabolic syndrome-associated HFpEF. According to Kondo et al. (63), the inhibition of SGLT1 increased the bioavailability of tetrahydrobiopterin (BH4) through an SGLT1/AMPK/Rac2-dependent mechanism, resulting in the improved coupling of NOS in primary human cardiomyocytes and myocardial tissues. Li et al. (64) reported a decrease in the myocardial infarct size, necrosis, and oxidative stress in transgenic mice with cardiomyocyte-specific RNA interference that knocked down SGLT1. The above examples suggest that part of the cardiovascular protection demonstrated by SGLT2 inhibitors may be attributed to the non-selective inhibition of SGLT1 receptors on cardiomyocytes.

## 7 Ventricular remodeling

The cardiac and pericoronary antifibrotic effects of SGLT2 inhibitors have been extensively studied using rodent models of diabetes. These beneficial effects do not appear to be associated with changes in blood pressure or glycemic control. Takasu et al. (65) conducted histopathological experiments using a nondiabetic DS/obese rat model of cardiomyopathy. They did not find any effect of ipragliflozin on the heart rate even after 6 weeks of treatment. However, ipragliflozin reduced the SBP, decreased the septal thickness, and ameliorated cardiomyocyte hypertrophy. In contrast to controls, the ipragliflozin treatment altered the expression profile of miRNAs associated with cardiac hypertrophy and heart failure in the left ventricle. These findings suggest that SGLT2 inhibitors may exert cardioprotective effects by altering miRNA expression profiles in patients with nondiabetic cardiovascular disease. Li et al. (66) demonstrated that empagliflozin attenuated cardiac remodeling without altering fasting glucose. In addition, TAC-treated mice demonstrated a 68.9% increase in their left ventricular mass, which was significantly attenuated by empagliflozin. Moreover, empagliflozin treatment reduced the TAC-induced increased collagen deposition (shown by Masson staining) by 54.2%. Transthoracic echocardiographic measurements showed that the ejection fraction (EF) and left ventricular shortening fraction (FS) were reduced by 55.3% and 60.1%, respectively. Following TAC, these changes were significantly attenuated with empagliflozin treatment, suggesting an improvement in cardiac contractile function. Compared with the sham-operated group, TAC elevated the isovolumic diastolic time (IVRT) and myocardial work done/activity index (MPI) by 85.9% and 100.6%, respectively. However, these changes in mice can be reversed to a great extent by empagliflozin treatment. Although engeletin did not reduce the elevated left ventricular end-systolic volume (LVESP) after TAC, it significantly reduced the left ventricular relaxation time constant (Tau) and end-diastolic pressure-volume relationship (EDPVR), suggesting improved diastolic function and increased LV compliance. Empagliflozin also reduced cardiac fibrosis in TAC-induced HF. Other SGLT2 inhibitors, such as dagliflozin and cargliflozin, can also reduce tissue fibrosis (67). Therefore, it can be hypothesized that SGLT2 inhibition is independent of hyperglycemia and has a direct and beneficial effect on the phenotypes and functions of cardiac fibroblasts.

## 8 Conclusion

**Figure 1:**
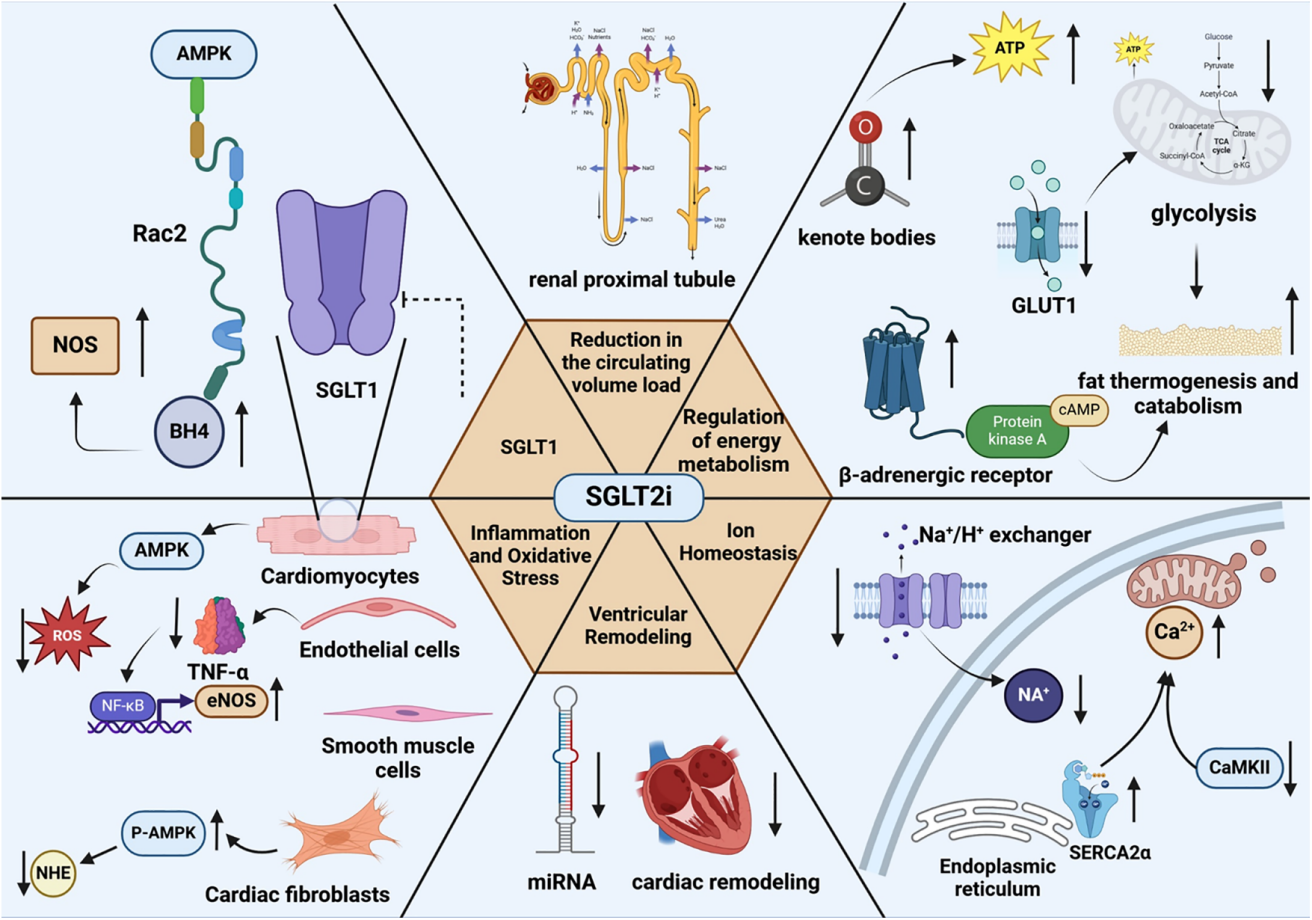
The mechanisms of SGLT2 inhibitors alleviate heart failure-related Non-hypoglycemic. SGLT1, sodium-glucose cotransporter-1; AMPK, AMP-activated protein kinase; Rac2, Rac family small GTPase 2; NOS, nitric oxide synthase; BH4, tetrahydrobiopterin; ATP, adenosine triphosphate; ROS, reactive oxygen species; TNF-α, tumor necrosis factor-α; NF-κB, nuclear factor kappa-B; eNOS, endothelial nitric oxide synthase; NHE, Na^+^/H^+^ exchanger;C aMKII, calcium/calmodulin-dependent protein kinase II; SERCA2α, sarcoplasmic/endoplasmic reticulum Ca^2+^–ATPase 2α. Created with biorender.com.

SGLT2 inhibitors, a novel insulin-independent hypoglycemic agent, can reduce hyperglycemic toxicity by lowering the glucose concentration. In addition (Figure 1), they can improve cardiac function in patients with heart failure by decreasing the circulating volume load, regulating energy metabolism, maintaining ionic homeostasis, mitigating inflammation and oxidative stress, directly inhibiting cardiac SGLT1 receptors, and enhancing antimyocardial fibrotic effects. Hence, these effects provide a sound theoretical basis for using SGLT2 inhibitors in non-T2DM patients to prevent or alleviate heart failure. However, most of the current evidence relies on animal studies, and the sample size of relevant clinical study populations is small and the diversity is limited. There is an urgent need to address these limitations in order to gain a deeper understanding of the role of SGLT2 inhibitors in the treatment of heart failure in a wider range of patient populations. It is in this context that the present review provides the relevant theoretical rationale and direction for this area of research.
